# Helicobacter Pylori e Pressão Alta

**DOI:** 10.36660/abc.20210629

**Published:** 2021-10-06

**Authors:** Andre Rodrigues Durães

**Affiliations:** 1 Universidade Federal da Bahia SalvadorBA Brasil Universidade Federal da Bahia, Salvador, BA - Brasil

**Keywords:** Hipertensão, Helicobacter pylori

A pressão arterial alta (PA) continua sendo a principal causa de morte no mundo, respondendo por 10,4 milhões de mortes por ano.^1^ Além disso, data da Organização Mundial da Saúde destacou o impacto global significativo da hipertensão arterial sistêmica (HAS): estima-se que 1,13 bilhão de pessoas em todo o mundo têm hipertensão, a maioria (dois terços) vivendo em países de baixa e média renda; e é uma das principais causas de morte prematura em todo o mundo.

A PA é determinada por vários parâmetros do sistema cardiovascular, incluindo volume sanguíneo e saída cardíaca (a quantidade de sangue bombeada pelo coração por minuto) e o equilíbrio do tom arterial que é afetado pelo volume intravascular e os sistemas neurohumorais.^2^

A HAS é uma elevação crônica da pressão arterial que, a longo prazo, causa danos nos órgãos alvo e resulta em aumento da morbidade e mortalidade. O aumento da resistência vascular sistêmica, da rigidez vascular e da responsividade vascular aos estímulos são centrais para a fisiopatologia da hipertensão.^3^ Além disso, a HAS é uma condição multifatorial que depende de fatores genéticos (epigenéticos), ambientais e sociais associados a fatores de risco metabólicos para doenças cardiocirculatórias e renais, tais como dislipidemia, obesidade abdominal, intolerância à glicose e diabetes mellitus.^4^

Entre as diversas possibilidades de associações já levantadas como relacionadas à HAS, Huang et al.^5^ trazem uma hipótese presente principalmente na literatura asiática a qual seria a associação entre Helicobacter pylori e a prevalência de HAS. Nesta metanálise, foram incluídos 17 estudos observacionais (11 de controle de caso e 6 transversais) de 1996 a 2019, totalizando 17.226 participantes. A detecção de H. pylori foi de 64,9% vs. 56,3% entre hipertensos e normotensos. Também detectaram uma relação estatisticamente significante entre H. pylori e HAS (razão de chances 2,07; intervalo de confiança de 95% (IC), 1,46, 2,94), que só permaneceu na população asiática. Recentemente, Xiong et al.^6^ publicou um estudo transversal chinês com 17.100 participantes e descobriu que indivíduos com infecção por H. pylori apresentaram maior prevalência de hipertensão arterial (57,5% vs. 55,1%, p = 0,002). A taxa de infecção por H. pylori em pacientes com hipertensão é maior do que em indivíduos não hipertensos (48,8% vs. 46,4%, p = 0,002). Após ajuste para potenciais fatores de confusão, a infecção por H. pylori aumentou a prevalência de hipertensão arterial (razão de chances, 1.117, IC95%, 1.029-1.213, p = 0,008). Este artigo agrega valor à hipótese dos autores. No entanto, podemos ver que temos informações baseadas em estudos observacionais, o que impossibilita a criação de uma relação causa-efeito.

A busca por fatores que possam estar causalmente relacionados à doença começa com a ideia de que as pessoas que têm a exposição devem ter uma frequência diferente da doença daqueles que não têm a exposição. Fator de confusão é o viés resultante da presença de causas comuns de exposições e desfechos. A confusão ocorre quando uma variável está associada tanto à exposição quanto à doença que estamos estudando.^7 - 9^ Determinar se uma variável é um fator de confusão requer fazer suposições intestáveis. O único progresso real que pode ser feito com o teste empírico para fatores de confusão é fazer outras suposições não testáveis que logicamente implicam em um teste para suposições que nos importam.^10^

Os autores têm o mérito de coletar sistematicamente todas as informações disponíveis e realizar uma metanálise sobre uma associação inesperada. Além disso, o desafio de buscar mais informações sobre a HAS é extremamente relevante devido ao impacto bem conhecido dessa condição em desfechos significativos nos níveis cardiovascular, renal e neurológico, uma vez que a HAS tem uma alta prevalência mundial. No entanto, o cuidado e a cautela são necessários ao utilizar dados de estudos observacionais que fornecem mais informações geradoras de hipóteses do que dados conclusivos. Portanto, é necessário realizar estudos prospectivos, preferencialmente randomizados, para elucidar essa hipótese.
