# Supplementary material for: Helicobacter Pylori e Pressão Alta
Source: Arq Bras Cardiol. 2021 Oct 6;117(4):637–8. [Article in Portuguese] doi: 10.36660/abc.20210629 (PMC8528362; doi:10.36660/abc.20210629)
Supplement: Translation

# Translation: Helicobacter Pylori and High Blood Pressure

**Authors:** Andre Rodrigues Durães

**Affiliations:** 1 Brazil Universidade Federal da Bahia, Salvador, BA - Brazil

**Keywords:** Hypertension, Helicobacter pylori

Raised blood pressure (BP) remains the leading cause of death globally, accounting for 10.4 million deaths per year.^1^ In addition, data from the World Health Organization highlighted the significant global impact of systemic arterial hypertension (SAH): an estimated 1.13 billion people worldwide have hypertension, most (two-thirds) living in low- and middle-income countries; and is a major cause of premature death worldwide.

BP is determined by several parameters of the cardiovascular system, including blood volume and cardiac output (the amount of blood pumped by the heart per minute) and the balance of arterial tone that is affected by intravascular volume and neurohumoral systems.^2^

SAH is a chronic elevation of blood pressure that, in the long-term, causes end-organ damage and results in increased morbidity and mortality. Increased systemic vascular resistance, vascular stiffness, and vascular responsiveness to stimuli are central to the pathophysiology of hypertension.^3^ Furthermore, SAH is a multifactorial condition that depends on genetic (epigenetic), environmental and social factors associated with metabolic risk factors for cardiocirculatory and renal systems diseases, such as dyslipidemia, abdominal obesity, glucose intolerance, and diabetes mellitus.^4^

Among the various possibilities of associations already raised as related to SAH, Huang et al.^5^ bring a hypothesis present in mainly Asian literature that would be the association between Helicobacter pylori and the prevalence of SAH. In this meta-analysis, they included 17 observational studies (11 case-control and 6 cross-sectional) from 1996 to 2019 with a total of 17226 participants. Detection of H. pylori was 64.9% vs. 56.3% between hypertensive and normotensive individuals. They also detected a statistically significant relationship between H. pylori and SAH (odds ratio 2.07; 95% confidence interval (CI), 1.46, 2.94), which only remained in the Asian population. Recently, Xiong et al.^6^ published a Chinese cross-sectional study with 17,100 participants and found that individuals with H. pylori infection had a higher prevalence of hypertension (57.5% vs. 55.1%, p = 0.002). The infection rate of H. pylori infection in patients with hypertension is higher than that in non-hypertensive individuals (48.8% vs. 46.4%, p = 0.002). After adjustment for potential confounders, H. pylori infection increased the prevalence of hypertension (odds ratio, 1,117, 95% CI, 1,029-1,213, p = 0.008). This paper adds value to the authors’ hypothesis. However, we can see that we have information based on observational studies, which makes it impossible to establish a cause-effect relationship.

The search for factors that might be causally related to disease begins with the idea that people who have the exposure should have a different frequency of the disease from those who do not have the exposure. Confounding is the bias resulting from the presence of common causes of exposures and outcomes. Confounding occurs when a variable is associated with both the exposure and the disease that we are studying.^7 - 9^ Determining whether a variable is a confounder requires making untestable assumptions. The only real progress that can be made with empirical testing for confounders is by making other untestable assumptions that logically imply a test for assumptions we care about.^10^

The authors have the merit of systematically collecting all available information and performing a meta-analysis on an unexpected association. Furthermore, the challenge of seeking more information about SAH is extremely relevant due to the well-known impact of this condition on significant outcomes at the cardiovascular, renal and neurological levels since SAH has a high worldwide prevalence. However, care and caution are necessary when using data from observational studies that provide more hypothesis-generating information than conclusive data. Therefore, it is necessary to carry out prospective studies, preferably randomized, to elucidate this hypothesis.
